# MXene in the lens of biomedical engineering: synthesis, applications and future outlook

**DOI:** 10.1186/s12938-021-00873-9

**Published:** 2021-04-01

**Authors:** Adibah Zamhuri, Gim Pao Lim, Nyuk Ling Ma, Kian Sek Tee, Chin Fhong Soon

**Affiliations:** 1 Biosensor and Bioengineering Lab, Microelectronics and Nanotechnology-Shamsuddin Research Centre, Institute for Integrated Engineering, Universiti Tun Hussein Onn Malaysia, Parit Raja, 86400 Batu Pahat, Johor Malaysia; 2 Faculty of Electrical and Electronic Engineering, Universiti Tun Hussein Onn Malaysia, Parit Raja, 86400 Batu Pahat, Johor Malaysia; 3 Faculty of Science and Marine Environment, Universiti Malaysia Terengganu, 21030 Kuala Nerus, Terengganu, Malaysia

**Keywords:** MXene, Biomedical application, Biosensors, Cancer theranostics, Drug delivery, Antimicrobial activity

## Abstract

MXene is a recently emerged multifaceted two-dimensional (2D) material that is made up of surface-modified carbide, providing its flexibility and variable composition. They consist of layers of early transition metals (M), interleaved with *n* layers of carbon or nitrogen (denoted as X) and terminated with surface functional groups (denoted as T_x_/T_z_) with a general formula of M_n+1_X_n_T_x_, where *n* = 1–3. In general, MXenes possess an exclusive combination of properties, which include, high electrical conductivity, good mechanical stability, and excellent optical properties. MXenes also exhibit good biological properties, with high surface area for drug loading/delivery, good hydrophilicity for biocompatibility, and other electronic-related properties for computed tomography (CT) scans and magnetic resonance imaging (MRI). Due to the attractive physicochemical and biocompatibility properties, the novel 2D materials have enticed an uprising research interest for application in biomedicine and biotechnology. Although some potential applications of MXenes in biomedicine have been explored recently, the types of MXene applied in the perspective of biomedical engineering and biomedicine are limited to a few, titanium carbide and tantalum carbide families of MXenes. This review paper aims to provide an overview of the structural organization of MXenes, different top-down and bottom-up approaches for synthesis of MXenes, whether they are fluorine-based or fluorine-free etching methods to produce biocompatible MXenes. MXenes can be further modified to enhance the biodegradability and reduce the cytotoxicity of the material for biosensing, cancer theranostics, drug delivery and bio-imaging applications. The antimicrobial activity of MXene and the mechanism of MXenes in damaging the cell membrane were also discussed. Some challenges for in vivo applications, pitfalls, and future outlooks for the deployment of MXene in biomedical devices were demystified. Overall, this review puts into perspective the current advancements and prospects of MXenes in realizing this 2D nanomaterial as a versatile biological tool.

## Introduction

Two-dimensional (2D) materials are currently of keen interest to material researchers due to their excellent electronic, mechanical, and optical properties. On the contrary, during early twentieth century, classical physicists predicted that the stability of 2D materials were thermodynamically ambiguous at any fixed temperature due to thermal lattice fluctuations [1]. Nevertheless, 2004 marked the scientific breakthrough in the world of material science with the discovery of 2D graphene monolayer [1]. Physically, 2D materials are atomically thin crystalline solids, bonded by covalent and van der Waals (vdW) bonding, since the discovery of graphene, more 2D materials such as boron nitride, metal oxides and chalcogenides have emerged from exfoliation of their respective 3D precursors [1, 2]. 2D nanomaterials are composed of layers with atomic- to nano-scale thicknesses, and they exhibit different novel properties compared to their 3D counterparts [3]. However, previous 2D nanomaterials are mainly used for fundamental and academic research [4]. Only just recently new materials have been introduced with greatly enhanced physical and chemical properties suitable for various research field [3, 5]. Electronic engineers are testing for energy storage systems and sensors [6, 7], and in more recent reports, they have been applied in bacterial cells and human cancer cells studies [8, 9].

**Fig. 1:**
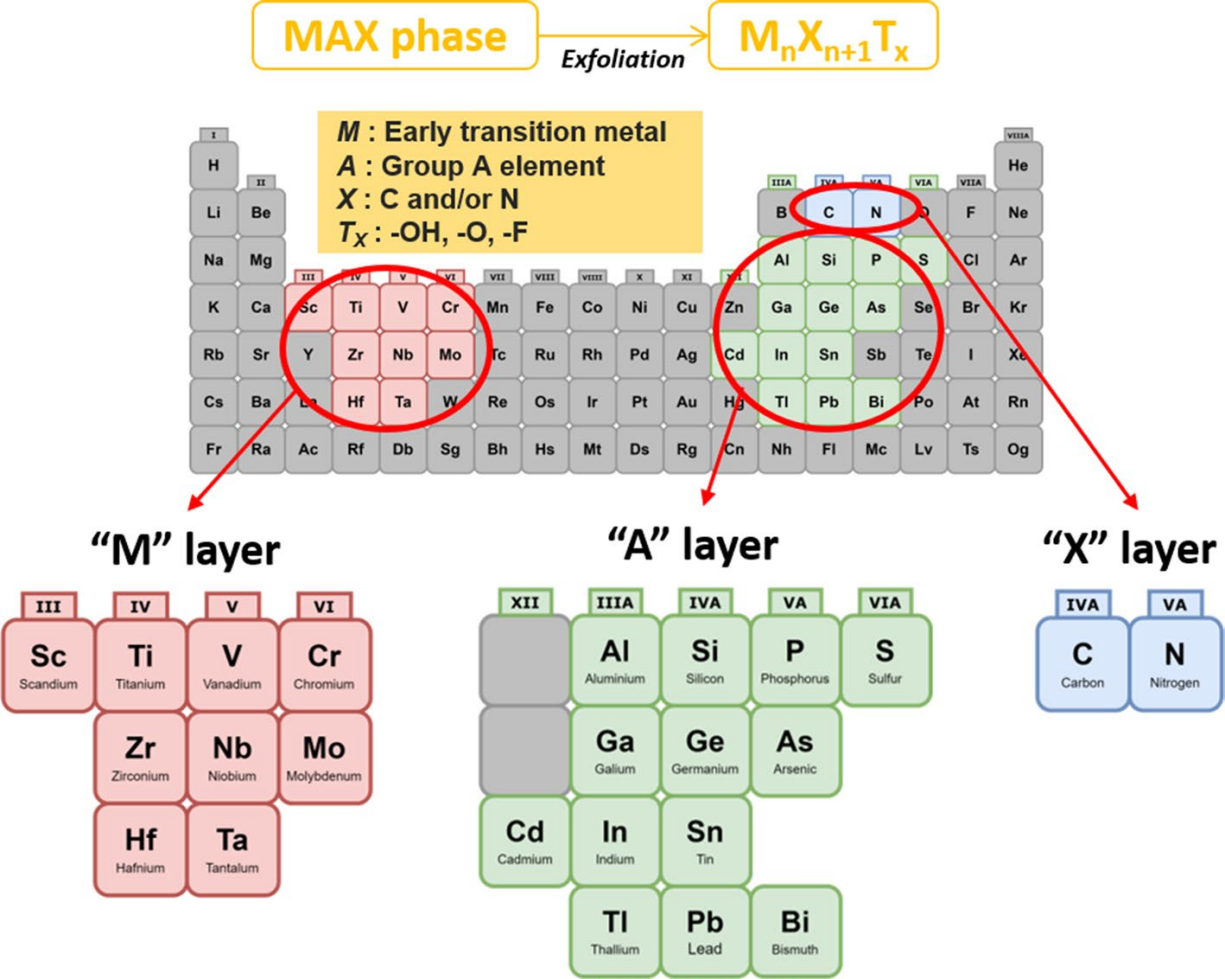
General element composition of MAX phase and MXene: M: early transition metal, A: Group A element, X: C and/or N, T_x_: surface functional group

"MXene" has emerged and acquired huge interest amongst the 2D nanomaterials research due to their modifiable chemical structures and exclusive characteristics. The first ever MXene was discovered by a group of researchers from Drexel University, Philadelphia, where they exfoliated 3D titanium aluminium carbide (Ti_3_AlC_2_) or known as MAX phase using hydrofluoric acid (HF), and produced 2D titanium-carbide (Ti_3_C_2_) layers [10]. MXenes are generally derived from transition metal carbides and nitrides [5]. MXenes, akin to graphene, are generally made from exfoliating their 3D precursors; this method is classified as a top-down approach. The 3D precursors for MXenes are called MAX phases, which are ternary carbides or nitrides with the general formula of M_n+1_AX_n_. As shown in Fig. 1, M is an early transition metal, A is an A-group element (mostly main group IIIA or IVA), X is either carbon or nitrogen, and *n* = 1, 2 or 3 [3]. Because M–X bonds are much stronger than M–A bonds, and the A layers are chemically more active than *M*–*X* layers, therefore, A layers can be selectively removed by a strong acid (i.e. hydrofluoric acid, HF) etching to produce M_n+1_X_n_ layers that can be further separated by sonication [6]. Through this etching process, the surfaces of MXenes are typically terminated with fluorine (–F), hydroxide (–OH) and oxygen (–O) groups due to its high surface energy [3]. Therefore, the final chemical formula of MXene is summarised as M_n+1_X_n_T_x_, where T_x_ is the surface functional groups [6]. Till date, the most applied MXenes for biomedical and biotechnology are from the Ti_3_C_2_ and Ti_2_C groups. This opens up the opportunity to have new combinations of the M and A groups of elements other than Ti and C.

MXenes are characterised by numerous properties that are useful in their fundamental aspects such as structural, optical, electronic and even biological properties [11]. These properties enable them for a broad application, with the most recent one being in the biomedical field. The specific characteristics of MXenes include high surface area, presence of hydrophilic functional groups, high atomic numbers (for certain transition metals) and paramagnetic behaviour [11–14]. The functional groups on MXenes also contribute to their rigidness and flexibility, which is quite important in thin film formation as a part of bio-electronic devices [15]. MXene nanosheets exhibit high surface area, hence they are suitable for drug loading and delivery for theranostics applications and synergistic disease treatment [13, 16]. The presence of hydrophilic functional groups is also important for drug loading/delivery, as the modification or functionalization increase biocompatibility for living cell/tissues [12]. The MXenes, consisting of high-atomic-number and paramagnetic transition metals, are more fitting for biomedical imaging, because they exhibit good X-ray attenuation for computed tomography (CT) scans and can be used as a magnetic resonance imaging (MRI) contrast agent [13]. Various types of MXenes could be synthesised based on different approaches, therefore, the biocompatibility assessment of MXene-based biomaterials is of utmost essential for overall biomedical applications.

**Fig. 2:**
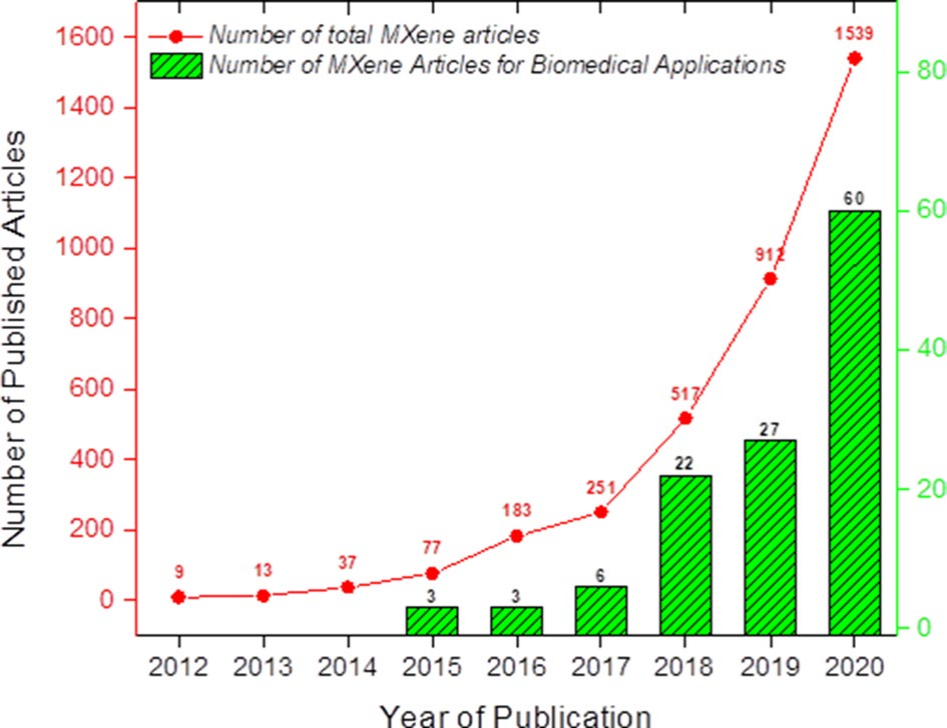
Number of journal publications on MXenes for biomedical applications, searched with keywords “MXene” and “Biomedical” ( Source: lens.org, 2012 to 2020)

For the past 6 years, there were a total of 121 publications on MXenes for biomedical applications reported in lens.org, searched with keywords “MXene” and “Biomedical” (Fig. 2). This amount of publication is relatively low for biomedical engineering compared to other well-known applications in electronics, catalysts and energy storage, which indicates that full potential of MXenes in biomedical applications remains scarce. Although research based on MXenes had been published since early 2012, MXene research on biomedical applications were only published 3 years later, since the first 3 years were dedicated to fundamental studies of MXenes, such as characterization of MXene structures to determine their properties. Research and studies on 2D MXenes for various types of biomedical applications are increasing, hence a comprehensive review of MXenes for these applications is important for an overview and guidance for future research. The aim of this review is to highlight the key MXene synthesis technologies with top notch biomedical applications. The current challenges and future outlooks of biocompatible MXenes are also discussed. This review will be useful as a guidance for upcoming new research of MXenes for more advanced and diverse applications.

## Review methodology

A systematic literature search was done to analyse published MXene papers from January 2015 to December 2020. Based on pre-selected criteria, appropriate research articles were selected using “lens.org”. The metadata obtained from Lens.org are integrated from various sources that include PubMed, Crossref, Microsoft Academic, CORE, ORCID and PubMed Central. Relevant and recent articles are needed to justify the proposed contents and maintain the originality of this review. Initial screening of relevant literature included searching of articles related to the keywords of this review, i.e., MXene, biomedical application, biosensors, cancer theranostics, drug delivery, antimicrobial activity. The literature search results are summarized in Table 1. After reviewing and analysing suitable articles, the overall sections of the review were gathered and deliberated with relevant examples.

**Table 1:** Literature search results collected from lens.org

Search terms	Number of journal articles published according to year						Total articles
	2015	2016	2017	2018	2019	2020	
“MXene”, “Biomedical”	3	3	6	22	27	60	121
“MXene”, “Biosensors”	2	2	1	7	17	30	59
“MXene”, “Cancer theranostics”	–	–	1	3	2	3	9
“MXene”, “Drug delivery”	–	–	2	4	1	9	16
“MXene”, “Antimicrobial”	1	–	–	7	2	8	18

## The Two Approaches for Synthesis MXene

In general, MXenes can be synthesised by either bottom-up or top-down approaches. Choosing the appropriate approach is crucial to determine their overall physical and chemical properties, such as size, morphology and functionality of the material [17]. After synthesis (regardless of bottom-up/top-down), MXenes surface can be further modified to enhance the biocompatibility/reduce the cytotoxicity of the material for biomedical applications.

### Top-down approach

**Fig. 3:**
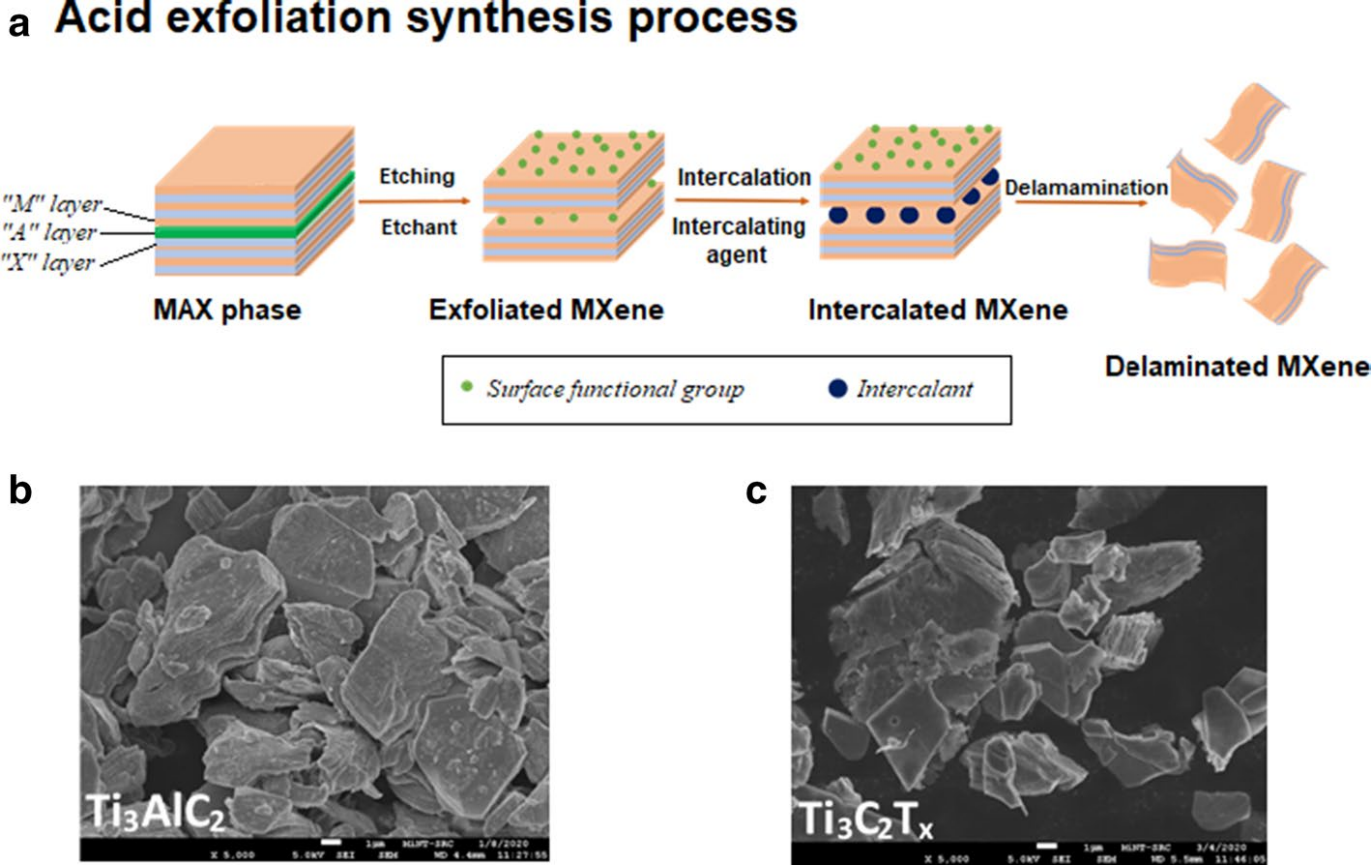
**a** Schematic illustration of top-down MXene synthesis process (acid exfoliation method) based on the descriptions in [20], **b** FE-SEM images of Ti_3_AlC_2_ MAX phase and **c** Ti_3_C_2_T_x_ MXene. FE-SEM images were obtained from Microelectronics and Nanotechnology-Shamsuddin Research Centre, Universiti Tun Hussein Onn Malaysia

The popular preparation method of MXene layers is the top-down fabrication approach initiated from MAX phase, which involves the acid exfoliation of layered A solids (Fig. 3). This method is considered as a classical method since the layered structure of MXene is similar to the structure of its 3D counterpart [14]. There are generally two steps: etching or cleavage of the MAX phase, and the delamination of the exfoliated MXene layers. For the synthesis of MAX phase, typically pristine powders of M, A and X in particular atomic ratios are mixed and heated to extreme high temperatures (~ 1200–1600 °C) [18]. The mixed sample will then be hot/cold pressed to densify the material and reduce microvoids or cracks in the material [19]. The pressure applied (~ 25–45 MPa) during the hot/cold pressing will determine the grain growth orientation. One issue with this method is that excessive A element powder (e.g. Si or Al) is needed, since this powder easily evaporates and sucked into the vacuum/argon (Ar) atmosphere at high temperatures [17]. Another common method of MAX phase synthesis is by pressure-less sintering, where the combined MAX powders were simply heated to a certain temperature [2, 18]. Pressure-less sintering was reported to produce highly oriented Ti_3_AlC_2_ MAX phase, compared to hot-pressed Ti_3_AlC_2_ [2]. However, this sintering limits the growth orientation of final MAX phase solids [17].

**Fig. 4:**
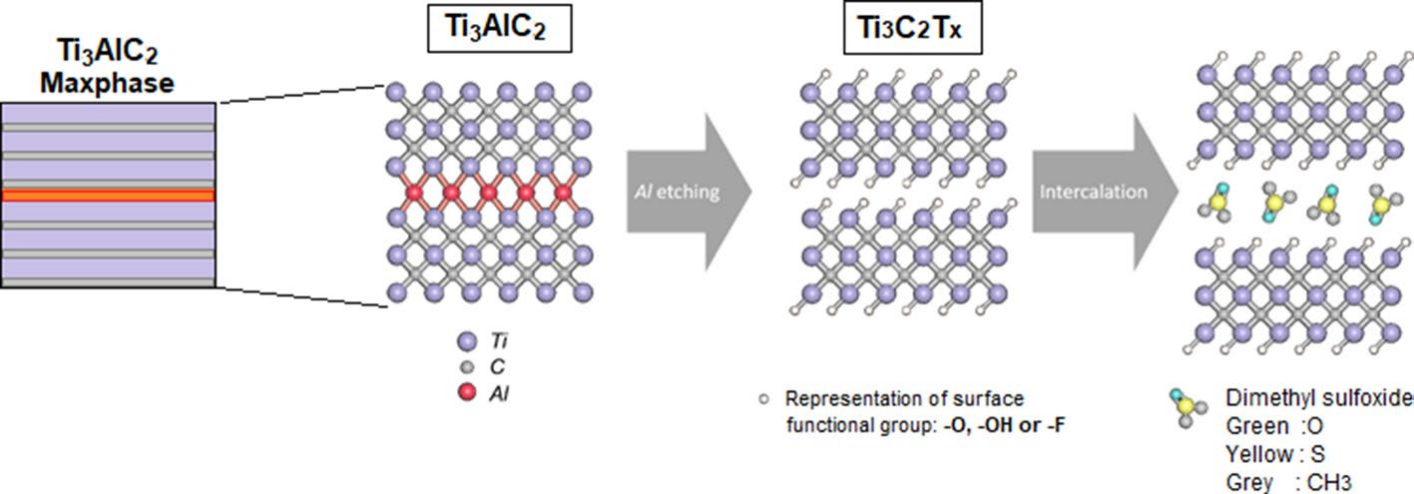
A schematic chemical representation of top-down approach: etching and intercalation procedure of MAX phase (Ti_3_AlC_2_) to form Ti_3_C_2_ MXene. The schematic is readapted with permission from [10]. Copyright (2011), Wiley–VCH

After the MAX phase is synthesised, the first synthesis step involves the etching of the 3D MAX phase such as Ti_3_AlC_2_ using a powerful etchant, usually HF [10]. M–A bonds are generally weaker than M–X bonds, hence selective etching of the M–A bonds is possible [25]. To etch the A—element from a common MAX phase, high concentration of fluoride ions (F−) must be involved, this is because F− ions bind strongly to the A—element (or Al) [25]. Additionally, HF treatment of the MAX phase results in different surface terminations with –OH, –O and –F groups as shown in Fig. 4 [21]. Chemical Eqs. (1), (2) and (3) show the general chemical reactions of MAX phase (M_3_AX_2_) with HF (aluminium was chosen as the A-layer) [10].

1 $${\text{M}}_{{{\text{n}} + {1}}} {\text{AlX}}_{{\text{n}}} \left( {\text{s}} \right) \, + {\text{ 3 HF }}\left( {{\text{aq}}} \right)\to {\text{M}}_{{{\text{n}} + {1}}} {\text{X}}_{{\text{n}}} \left( {\text{s}} \right) \, + {\text{ AlF}}_{{3}} \left( {{\text{aq}}} \right) \, +\frac{3}{2} {\text{H}}_{{2}} \left( {\text{g}} \right)$$

2 $${\text{M}}_{{{\text{n}} + {1}}} {\text{X}}_{{\text{n}}} \left( {\text{s}} \right) \, + {\text{ 2 HF }}\left( {{\text{aq}}} \right)\to {\text{M}}_{{{\text{n}} + {1}}} {\text{X}}_{{\text{n}}} {\text{F}}_{{2}} \left( {\text{s}} \right) \, + {\text{ 2 H}}_{{2}} \left( {\text{g}} \right)$$

3 $${\text{M}}_{{{\text{n}} + {1}}} {\text{X}}_{{\text{n}}} \left( {\text{s}} \right) \, + {\text{ 2 H}}_{{2}} {\text{O }}\left( {{\text{aq}}} \right)\to {\text{M}}_{{{\text{n}} + {1}}} {\text{X}}_{{\text{n}}} \left( {{\text{OH}}} \right)_{{2}} \left( {\text{s}} \right) \, + {\text{ H}}_{{2}} \left( {\text{g}} \right).$$

Previous studies as summarised in Table 2 have reported HF etching of MAX phases using different concentrations of HF with different immersion times and temperature [10, 16, 22, 23]. The very first MXene synthesised by Naguib et al*.* involved immersing Ti_3_AlC_2_ powders in 50% concentrated HF at room temperature for 2 h to yield full dissociation of the MAX phase [10]. Other examples include Ti_3_C_2_ MXene production using 48% HF-water and stirred at room temperature for 24 h [22], and Ti_3_C_2_ multi-layered nanosheet production by stirring in 40% HF at 45 °C for 2 days [16].

**Table 2:** Etching and intercalant conditions of MXene (top-down approach) synthesis as reported by several key studies

MAX phase(s)	Etchant (conditions)	Intercalant (conditions)	Ref
Ti_3_AlC_2_	50% HF (2 h, RT)	–	[10]
Ti_3_AlC_2_	48% HF (24 h, RT)	TMAOH (24 h, RT)	[22]
Ti_3_AlC_2_	40% HF (2 days, 45 °C)	TMAOH (3 days, 45 °C)	[16]
Ti_3_AlC_2_, Ti_2_AlC_,_ Nb_2_AlC	LiF + HCl (45 h, 40 °C)	–	[23]
Ti_3_AlC_2_	LiF + HCl (24 h, 50 °C) NH_4_F + HCl (24 h, 30 °C) KF/NaF + HCl (48 h, 40 °C)	– – –	[24]
Ti_2_AlC	LiF + HCl (48 h, 50 °C) NH_4_F/KF + HCl (48 h, 40 °C) NaF + HCl (24 h, 60 °C)	– – DMSO (24 h), NH_3_.H_2_O (2 h) or urea (24 h, 60 °C)	
Ti_3_AlC_2_	NH_4_HF_2_ (10–160 min, RT)	–	[25]
Ti_3_AlC_2_	NaOH (12 h, 270 °C)	–	[27]
Ti_4_AlN_3_	Mixture of LiF, KF, NaF (30 min, 550 °C)	TBAOH (5 min)	[28]
Ti_3_AlC_2_	–	DMSO (18 h, RT)	[31]
Nb_2_AlC	50% HF (48 h, 55 °C)	Isopropylamine (18 h, RT)	[32]
V_2_AlC Ti_3_AlCN	48% HF (92 h, RT) 30% HF (18 h, RT)	TBAOH (4 h, RT)	[33]
Ti_3_AlC_2_	40% HF (3 days, RT)	TPAOH (3 days, RT)	[34]

Another most widely used etching method involves in situ formation of HF, where the reaction of an acid (e.g. hydrochloric acid, HCl) and a fluoride salt forms upon HF mixing [13]. This method promotes safer etching of aluminium (Al) and avoids the harmful effects of concentrated HF. The first study on fluoride salt etching of MAX phase was using solution of lithium fluoride (LiF) and HCl with heating at 40 °C for 45 h [23]. This method successfully generated other MAX phases such as Nb_2_AlC and Ti_2_AlC, proving that this method is versatile to multiple MAX phases. Ti_3_C_2_ and Ti_2_C nanosheets can also be generated through etching of various fluoride salts such as lithium fluoride (LiF), sodium fluoride (NaF), potassium fluoride (KF) and ammonium fluoride (NH_4_F) in HCl with optimum temperature, resulting in MXene that can store methane at atmospheric pressure and absorb methane under high pressure [24]. Another study was done where Ti_3_AlC_2_ films were etched using only ammonium bifluoride (NH_4_HF_2_), without using any harsh acid [25]. They found that during the etching process, the MXene layers were simultaneously intercalated with the ammonium species (NH_4_^+^), producing layers with larger lattice parameter (> 25%) compared to HF-etched Ti_3_C_2_.

Although many studies still employ HF/fluoride source as the preferred MAX phase etchant, this acid is very toxic and is very dangerous to handle, especially for biological applications, because even a tiny amount of unreacted HF could induce cell death [14]. In humans, HF can cause systemic toxicity that can lead to fatality [26]. Therefore, direct use of HF, or even in situ formation of HF poses safety and environmental hazards that hinders the advancement of MXenes’ application [26]. Another downside of this type of etching is the abundance of fluoride ions (F^−^) on the surface of MXene, which decrease the amount of other functional groups (–OH, –O). –OH and –O functional groups are easier to be functionalized, and it is challenging to conjugate F− ions [14]. Hence, a fluorine-free etching method is more favourable to produce MXenes with controllable functional surface termination for various biomedical applications. One study by Li et al*.* proposed and successfully fabricated multilayer Ti_3_C_2_T_x_ through alkali-assisted hydrothermal method using aqueous sodium hydroxide (NaOH) solution as the etchant, at a high temperature of 270 °C [27]. Via this method, they reported production of high-quality Ti_3_C_2_ powder with 92% purity with more –OH and –O terminations. Another method involves the use of molten salt, by mixing of Ti_4_AlN_3_ powder with molten fluoride salt (mixture of LiF, KF, and NaF of a specific ratio) at 550 °C for 30 min [28]. After delamination of the Ti_4_N_3_ layers with tetrabutylammonium hydroxide (TBAOH), multi-layered and single-layered MXenes were produced.

After etching, MXenes typically undergo delamination process to separate the MXene sheets so that the properties of MXenes’ 2D state can be investigated further [29]. The delamination step can be done by using intercalating agent/intercalant and sonication, where absence of intercalating agents yield smaller sized MXenes with larger defects [13, 29, 30]. Intercalating agents often increase the *c-*lattice parameter, or the distance between two consecutive MXene sheets, making them easier to separate/delaminate to form pure MXene sheets [29]. Examples of common intercalants used include dimethylsulfoxide (DMSO) and isopropylamine, which are polar organic solvents [31, 32] (Table 1). The delamination of Ti_3_C_2_ sheets using DMSO for 24 h at room temperature, resulted in increase of *c*-lattice parameter from 19.5 ± 0.1 Å to 35.04 ± 0.02 Å, indicating that the MXene sheets were successfully intercalated with DMSO [31]. The delamination of Nb_2_C (niobium carbide) MXenes for lithium energy storage devices were produced from isopropylamine as the intercalant for 18 h at room temperature, and the interlayer distance increased by ≈ 12.3 Å [32]. Other intercalants agents such as tetrabutylammonium hydroxide (TBAOH) or tetrapropylammonium hydroxide (TPAOH) were also reported [33]. The used of TBAOH for delamination of vanadium carbide (V_2_CT_x_) and titanium carbonitride (Ti_3_CNT_x_) induced huge impromptu swelling, and simultaneously weakened the interlayer interactions, therefore increasing the yields of delaminated MXene compared to using DMSO [33]. Ultrathin Ti_3_C_2_ (5–6 nm) was synthesised using a modified chemical exfoliation, by HF etching for 30 days followed by intercalation with TPAOH for another 3 days [34]. The average lateral size of the thin Ti_3_C_2_ flakes was around 150 nm, and the flakes also exhibited 2D sheet-like morphology upon intercalation with TPAOH.

It is crucial for the MXene nanosheets to be nano in size for biological applications. Traditional multi-layered MXenes, synthesised from top-down approach, produce large sheet sizes, that may lead to potential biosafety issues, and low therapeutic outcome. Also, large MXene sheets are not suitable for intravenous (IV) administration, because this usually requires nano-sized particles for easy transport in our circulatory system and good penetration and accumulation in cancer tissues [35]. Hence, based on the overall studies reported on top-down synthesis of MXene, the optimization of etchant, etching time, and type of intercalant are key parameters to ensure successful synthesis of biocompatible MXene nanosheets.

### Bottom-up approach

**Fig. 5:**
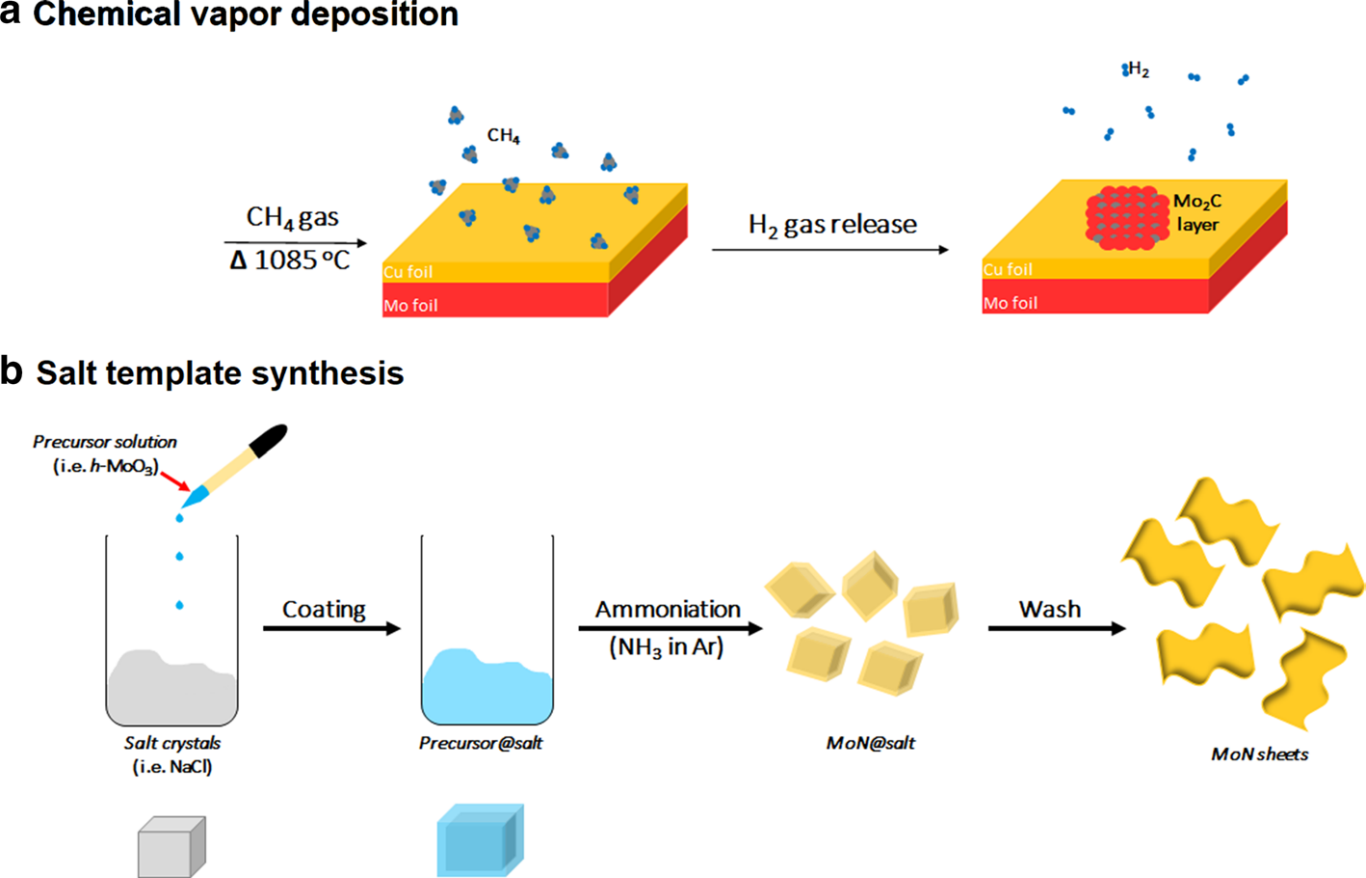
Schematic illustration of bottom-up methods. **a** Chemical vapour deposition of Mo and C to form Mo_2_C thin film in gas chamber based on the report in [37], **b** Salt-template synthesis of MoN nanosheets. Readapted with permission from [38]. Copyright (2017) American Chemical Society

Another lesser known MXene synthesis technique is the bottom-up synthesis approach, by atomic scale control [13]. Bottom-up synthesis commonly begins from small organic/inorganic molecules/atoms, followed by crystal growth that can be organized to form 2D-ordered layer [14]. The most common method for this approach is the chemical vapour deposition (CVD) technique, which can produce good quality of thin films on various substrates (Fig. 5a). In general, CVD produces very thin films that are often multi-layered (at least six layers) [36]. The first MXene synthesis by CVD technique produced high-quality ultrathin Mo_2_C (molybdenum carbide) using methane gas (CH_4_) as the carbon source and a Cu/Mo (copper/molybdenum) foil as the substrate at temperature above 1085 °C (Fig. 5a). The optimisation of growth temperature and growth time produced range of films with lateral sizes between 10 and 100 μm. The Mo_2_C films synthesized were defect-free and possessed high crystallinity, which may indicate the lack of surface functional groups [37]. This is unfortunately not ideal for biomedical applications, as well-functionalized MXenes are preferred for the surface engineering of the nanosheets, and the size (μm) is too large for permeation of cells. Other than CVD, methods such as template method and plasma-enhanced pulsed laser deposition (PELPD) had also been explored for MXene synthesis [38, 39]. The first PELPD-synthesized ultrathin Mo_2_C films using methane plasma as a carbon source, reacted with Mo vapour which was generated by the pulsed laser. This reaction was done on a sapphire substrate, heated to 700 °C to produce high-quality films with film thickness that can be controlled by varying the laser pulse rate. Other modification such as the scalable salt-template synthesis of 2D nitrides (MoN, V_2_N and W_2_N) by reducing their respective 2D hexagonal oxides with ammonia was reported [35]. They found that salt-templating is capable of producing 2D metal oxide precursors, and the MoN nanosheets produced from this synthesis were hydrophilic and had sub-nm thickness when dispersed in water (Fig. 5b) [38]. This method is proven to be applicable on other metal oxides, such as V_2_N and W_2_N nanosheets. Nevertheless, only limited information of bottom-up synthesis of biocompatible MXene are available, provide room for improvement [29]. Another important aspect of both top-down and bottom-up syntheses is the robustness and safe methods for large-scale synthesis, which also needs to be further investigated. The main differences between the top-down and bottom-up synthesis approaches of MXene and the material properties discussed are as summarized in Table 3.

**Table 3:** Comparison of top-down and bottom-up synthesis approaches of MXene

	Top-down approach	Bottom-up approach	Refs.
Precursor	Initiate from MAX phase of 3D structure	Initiate from atom to crystal growth of MXene film	[10, 36]
Method	Involves with chemical etchants such as HF acid	Apply chemical vapour deposition or salt-templating method or plasma-enhanced deposition	[36–38]
Synthesis condition	Pressure-less synthesis	Control gas flow as carbon source	[10, 37]
Temperature	Require room or low temperature for synthesis	Require high temperature of ~ 1000 °C for synthesis	[36]
Morphology	Large irregular MXene sheet could be produced with a lateral size of a few hundred nanometers and thin sheets with a thickness between 10 and 200 nm	Produce defect-free and highly crystalline thin film of multilayers with a lateral size of between 10 and 100 μm	[22, 36]
Surface properties	Functionalized –OH and –O after synthesis	Lack of functional groups after synthesis	[3, 37]

## Biomedical applications of MXene

**Fig. 6:**
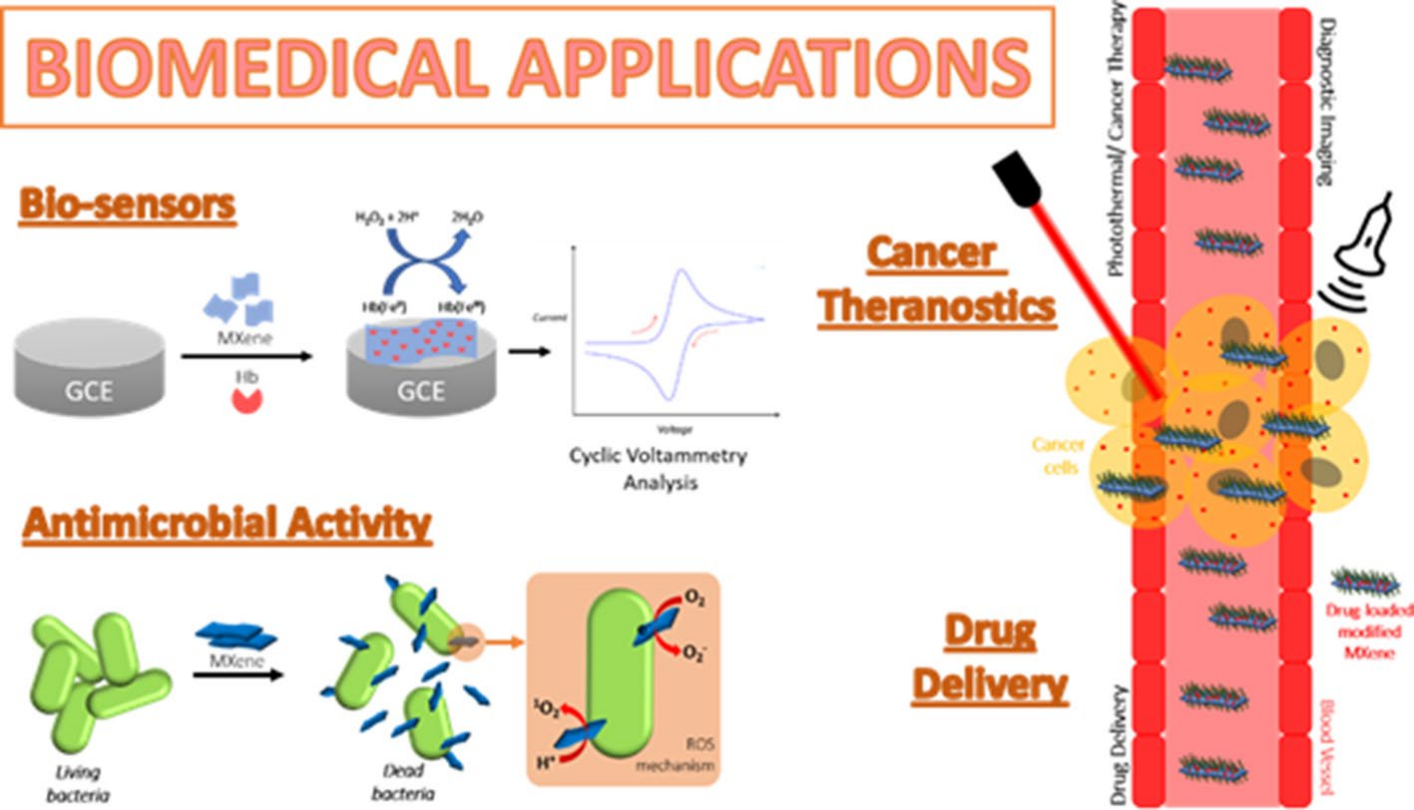
MXenes for biomedical applications: biosensors, cancer theranostics, drug delivery, antimicrobial activity. The figure is reillustrated based on the reports in [41–43]

MXenes exist as stacked 2D sheets, held together by weak van der Waals (vdW) forces and/or strong hydrogen-bonding interactions between the surface functional groups [40]. These surface functional groups are chemically reactive and can be further functionalized. In biomedical research studies, the surfaces of MXenes can be tuned with various materials suitable for biosensors, cancer theranostics (therapeutics and diagnostics), drug delivery, and antimicrobial activity (Fig. 6). The type of MXene composite and its application in biomedical are summarized in Table 4.

**Table 4:** Summary of types of MXenes, their syntheses methods, surface functionalization and biomedical applications. Note: LOD indicates the lowest level of detection within the detection range stated. LOD is the lowest concentration of a substance/analyte that can be detected by a reliable analytical method, whereas the detection range includes lowest to highest concentrations of substance that are able to be detected accurately and precisely

Type of MXene/MXene composite	Synthesis method of MXene	Functionalization(s)	Application	Application details	Ref
TiO_2_–Ti_3_C_2_	Hydrothermal synthesis	Hemoglobin (Hb), Nafion	Bio-sensor	Detection of hydrogen peroxide **(LOD: 14 nM; range: 0.1**–**380 μM)** and nitrite **(LOD: 0.12 μM; range: 0.1**–**380 μM**) via amperometry changes	[44, 45]
Au/Ti_3_C_2_	HF etching	Glucose oxidase (GO_x_)		Detection of hydrogen peroxide through oxidation of glucose **(LOD: 5.9 μM; range: 0.1–18 mM)**	[46]
Ti_3_C_2_	LiF + HCl etching	Poly-L-lysine (PLL), GO_x_		Detection of hydrogen peroxide through oxidation of glucose **(LOD: 2.6 μM; range: 4**–**20 μM, 0.02**–**1.1 mM)**	[47]
Ti_3_C_2_	HF etching	Tyrosinase, chitosan		Detection of phenol in real water samples **(LOD: 12 nM; range: 0.05**–**15.5 μM)**	[48]
Ti_3_C_2_	HF etching	β-hydroxybutarate dehydrogenase, bovine serum albumin (BSA), glutarate		Detection of β-hydroxybutarate **(LOD: 44.5 μM; range: 360 μM**–**17.91 mM)**	[49]
Ti_3_C_2_	LiF + HCl etching	(3-Aminopropyl) triethoxysilane (APTES), BSA, anti-CEA		Detection of carcinoembryonic antigen (CEA) **(LOD: 1.8 × 10**^**–5**^** ng mL**^**−1**^**; range: 0.0001**–**2000 ng mL**^**−1**^**)**	[50]
Ti_3_C_2_	HF etching, TMAOH intercalation	AuNPs, staphylococcal protein A, anti-CEA		Detection of CEA via surface plasmon resonance (SPR) **(LOD: 0.7 fM; range: 0.0002**–**20 000 pM)**	[51]
Ti_3_C_2_	HF etching	Hollow AuNPs, APTES, SPA, anti-CEA		Detection of CEA via SPR **(LOD: 0.15 fM; range: 0.001**–**1000 pM)**	[52]
Ti_3_C_2_	HF etching, DMSO intercalation	Osteopontin (OPN) aptamer, phosphomolybdic acid (PMo_12_), polypyrrole (PPy),		Detection of overexpressed OPN **(LOD: 0.98 fg mL**^**−1**^**; range: 0.005 pg mL**^**−1**^–**10 ng mL**^**−1**^**)**	[53]
Ta_4_C_3_	HF etching	Manganese oxide (MnO_x_), soybean phospholipid (SP)	Cancer theranostics	Multi-imaging-guided (MRI, CT scan and PAI) PTT	[54]
Ti_3_C_2_	HF etching, TPAOH intercalation	Manganese oxide (MnO_x_), soybean phospholipid (SP)		MRI-guided PTT	[55]
Ta_4_C_3_	HF etching	Supermagnetic iron oxide nanoparticles (IONPs)		MRI-guided PTT	[56]
Ti_3_C_2_	HF etching, TPAOH intercalation	Poly(lactic-*co*-glycolic acid) (PLGA), SP, IONPs		MRI-guided PTT	[34]
Nb_2_C	HF etching, TPAOH intercalation	Polyvinylpyrrolidone (PVP)		PAI-guided PTT	[57]
Nb_2_C	HF etching, TPAOH intercalation	Cetanecyltrimethylammonium chloride (CTAC), APTES, polyethylene glycol (PEG)		PAI-guided PTT	[58]
Ti_3_C_2_ QDs	Hydrothermal synthesis	–		Multicolour cellular imaging	[59]
Ti_3_C_2_ QDs	Sonication probing in TPAOH	–		PTT in NIR biowindow, biocompatibility test	[60]
Ti_2_N QDs	KF + HCl etching, sonication in NMP	SP		PAI-guided PTT	[61]
Nb_2_C QDs	HF etching, TPAOH sonication (ultrasound-assisted)	–		Fluorescence imaging, metal ions sensing	[62]
Ti_3_C_2_	HF etching, TPAOH intercalation	SP, doxorubicin (Dox)	Drug delivery	Chemotherapeutic agent, synergistic chemotherapy and PTT	[63]
Ti_3_C_2_	HF etching	Cellulose, Dox		Chemotherapeutic agent, synergistic chemotherapy and PTT	[64]
Ti_3_C_2_	HF etching	Polyacrylamide (PAM)		Study of drug release	[65]
Ti_3_C_2_	LiF + HCl etching	Cobalt nanowires (CoNWs), Dox		Study of drug release control, synergistic chemotherapy and PTT	[66]
Ti_3_C_2_	LiF + HCl etching	–	Antimicrobial activity	Study of antibacterial activity	[67]
Ti_3_C_2_	LiF + HCl etching	PVDF		For wastewater treatment	[68]
Ti_3_C_2_	LiF + HCl etching	Chitosan, glutaraldehyde		Study of antibacterial activity	[69]
Ti_3_C_2,_ Ti_2_C	HF etching	–		Comparison study of antibacterial activity	[70]
Ti_3_C_2_	HF etching	PLL		Study of antibacterial activity	[22]
Ti_3_C_2_	LiF + HCl etching	–		Study of antibacterial activity	[71]
Ti_3_C_2_	HF etching	–		Study of antifungal activity	[20]

### Biosensors

#### Enzyme-based biosensors

MXenes are well known for its high electrical conductivity (Ti_3_C_2_T_x_ monolayer: 4600 ± 1100 S/cm) [72], excellent ion transport behaviour, good biocompatibility, high surface area to volume ratio and easy to functionalize [73]. Hence, these properties allow MXenes to be recognized as a very advanced biosensing tool which can detect various small molecules, large biomolecules, and even cancer cells. Generally, for detection of small molecules, enzymes are immobilized on the MXene nanosheets to catalyse the chemical reaction of the molecules. An organ-like TiO_2_–Ti_3_C_2_ nanocomposite was synthesised via hydrothermal synthesis, and was fabricated with hemoglobin (Hb) to create a mediator-free biosensor that can detect hydrogen peroxide (H_2_O_2_) [44]. H_2_O_2_ is a reactive oxygen species (ROS) that is generated from aerobic metabolism, which regulates various biological processes at physiological amounts but very toxic at large amounts in the body [74]. MXenes are capable of H_2_O_2_ sensing, through the oxidation of their surface terminations by H_2_O_2_, and this reaction will increase the oxygen density on their surface and promote the charge transfer process [14]. The benefit of employing TiO_2_–Ti_3_C_2_ nanocomposite, or nanomaterials in general, is to facilitate the direct electron transfer (DET) while retaining the bioactivity of the immobilized enzymes [75]. The TiO_2_–Ti_3_C_2_ nanocomposite is a biocompatible matrix suitable for enzyme immobilization, with their biosensors having a low limit of detection (LOD) and wide linear range for H_2_O_2_ detection [44]. Moreover, the same system could be applied for the detection of nitrite (NO_2_^−^) with and even lower LOD and wider detection range [45].

MXenes have been fabricated for amperometric biosensor platform, based on Au/Ti_3_C_2_ nanocomposite for sensitive enzymatic glucose detection [46]. The glucose biosensor works by immobilizing glucose oxidase (GO_x_) to an appropriate electrochemical transducer, and further functionalized with gold (Au). The GO_x_ catalyses the oxidation of glucose to gluconolactone and H_2_O_2_, while the Au improves electron transfer process between the GO_x_ and Au-coated glassy carbon electrode (GCE). The Au/Ti_3_C_2_ nanocomposite biosensor yielded low LOD with wide linear range for glucose detection, with excellent stability and reproducibility. Modification of the Au/Ti_3_C_2_ nanocomposite with poly-L-lysine (PLL) increased the loading capacity by 39.5 wt% at pH 6.7, and by 31.7 wt% at pH 5.5 [47]. As the pH decreases, the charge density of PLL increase, providing a stronger electrostatic attraction between PLL and Ti_3_C_2_ nanosheets and GO_x_ molecules. The PLL-modified Au/Ti_3_C_2_ biosensor had a lower LOD and could detect glucose in both micro- and milli-Molar ranges [46]. A Ti_3_C_2_-based tyrosinase (Tyr) biosensor was fabricated for detection of phenol in real water samples [48]. Phenolic compounds are abundant in nature, and they tend to dissociate into other moieties that are very toxic upon entry in water [76]. Chitosan (Chi) was added to the biosensor to hinder direct electron transfer from the electrode itself. The tyrosinase biosensor was reported to have an ultra-low LOD in the nano-Molar scale, with high detection sensitivity of 414 mA M^−1^.

Modification of Ti_3_C_2_ nanosheets with β-hydroxyburate dehydrogenase enable the detection of β-hydroxyburate (β-HBA) [49]*.* Determination of β-HBA is important for humans, especially for patients with diabetic ketosis as the metabolic acidosis can occur when blood β-HBA levels reach up to 20 mM [77]. The Ti_3_C_2_-based β-HBA biosensor was fabricated using gold-printed circuit board (Au-PCB) as the electrode instead of the conventional GCE, and the overall performance of the biosensor was excellent, with low LOD and very wide detection range [49].

#### Detection of cancer biomarkers

Carcinoembryonic antigen (CEA) is generally used as tumor markers [51]. The first MXene-based CEA detector use single/few-layered Ti_3_C_2_ MXene coated with an amino groups of receptor (in this case 3-Aminopropyl triethoxysilane (APTES)) to covalently immobilize carcinoembryonic monoclonal antibody for cancer biomarker detection [50]. Glassy carbon electrode (GCE) was employed as the electrode, and BSA was used to block unspecified active site of the bioelectrode to complete the biosensing platform, resulting in an extremely low LOD and very wide linear detection range in the nano-scale with sensitivity of 37.9 µA ng^−1^ mL cm^−2^ per decade. To enhance the sensitivity of CEA biosensor, surface plasmon resonance (SPR) technology has been introduced, which enables the measurement of refractive-index and real-time interactions of biological and chemical molecules [52]. In this study, prepared Ti_3_C_2_ nanosheets were functionalized with AuNPs (gold nanoparticles) through a chemical reduction method [52]. The synthesized nanocomposite was further decorated with staphylococcal protein A (SPA) to immobilize the anti-CEA antibody (Ab_1_) onto the nanocomposite surface (labelled as Ti_3_C_2_ MXene/AuNPs/SPA). Another nanocomposite MWPAg-A_2_ synthesized from multi-walled carbon nanotubes–polydopamine–silver nanoparticles (MWCNTs–PDA–AgNPs) nanohybrids that conjugated with polyclonal anti-CEA antibodies (Ab_2_) to enhance the signal of the sensing system, which enables ultra-low LOD in the femto-scale and detection range in the pico-scale [51]. Ultrathin Ti_3_C_2_ was also employed on the sensing platform and signal enhancer of the SPR biosensor, which also exhibited an ultra-low LOD and wide detection range in pico-scale [78].

Apart from detecting CEA as tumour marker, overexpressed proteins such as osteopontin (OPN), which could lead to cancer progression have also been reported. OPN is a phosphoprotein that regulates tumour metastasis, and is commonly overexpressed in tumour stromal cells that may lead to progression of cancer [79]. Ti_3_C_2_T_X_-PMo_12_ (phosphomolybdic acid) nanohybrid was embedded with polypyrrole (PPy), as a platform to boost the anchoring of osteopontin (OPN) aptamer for OPN detection [53]. The PPy@Ti_3_C_2_T_X_/PMo_12_-based aptasensor consisted of Ti^4+^ and Mo^4+^ ions which were integrated within the MXene nanosheets and conjugated PPy matrix to facilitate the immobilization of the targeted OPN aptamer strands. This proposed aptasensor displayed ultra-low LOD in femto-scale, with a very wide linear concentration range of 0.05 pg mL^−1^–10.0 ng mL^−1^.

Based on both enzyme-based and cancer biomarkers biosensors studies, MXenes, especially Ti_3_C_2_T_X_, can immobilize various types of molecules for improved stability and performance of the biosensors. MXene composite is an advance biocompatible matrix suitable for mediator-free, direct electrochemical biosensing devices that have wide potential in bio-detection and environmental analyses.

### Cancer theranostics

#### MXene composites

2D nanomaterials have gained a lot of attention as the ultrathin nanostructure and beneficial physicochemical and biological properties fit for cancer theranostics application [80]. The combination of theranostics, therapeutic functionality and diagnostic imaging allow early diagnosis of cancer or any fatal illness for precision treatment [11, 81]. The surface of 2D nanomaterials can be functionalized with various molecules to activate the theranostics functions in the nanomaterials. Many studies on MXene composites as contrast agent for bio-imaging integrated with photothermal functions have been reported. The first study of MXene composite for cancer theranostics was using tantalum carbide (Ta_4_C_3_) nanosheets with manganese oxide (MnO_X_) that were integrated with soybean phospholipid (SP) to form a new material, MnO_X_/Ta_4_C_3_–SP composite [54]. MnO_X_/Ta_4_C_3_–SP composite is tested for photothermal tumour ablation, and each layer displayed different functionality. As such, MnO_X_ is for tumour microenvironment responsive magnetic resonance imaging (MRI), Ta is desirable for contrast-enhanced computer tomography (CT) scan [82, 83] and SP to stabilised the nanocomposite for physiological environments with low cytotoxicity [54, 55, 84]. This study proved that MnO_X_/Ta_4_C_3_–SP composite successfully achieved contrast-enhanced photoacoustic (PA) imaging and photothermal therapy (PTT) for tumour-growth suppression [54]. Moreover, using Mn outfit conventional Gd^3+^ (gadolinium)-based agents could result in nephrogenic systemic fibrosis (NSF) in high concentrations [82, 85] and Ta element has high atomic number (*Z* = 73) with high X-ray attenuation coefficient (Ta: 4.3 cm^2^ kg^−1^, Au: 5.16 cm^2^ kg^−1^ at 100 eV) [55, 83]. Since then, many studies have been focused on functionalizing MXenes as suitable PA and PTT agents. PA imaging is an emerging imaging modality that is non-invasive, and provides superb contrast, high spatial resolution and deep tissue penetration by detecting the acoustic wave that constructs PA images [84]. Most PA contrast agents can also be used as PTT agents; PTT is a therapeutic modality for thermal ablation of cancers, that has high ablation efficiency, causing minimal damage to normal tissues, and is normally triggered by near-infrared (NIR: > 750 nm) laser [86]. The MnO_x_/Ta_4_C_3_–SP composite reported a photothermal conversion efficiency (34.9%) that is greater than Au nanorods (21%) [87]. Ti_3_C_2_ MXene was also functionalized with MnO_x_ and SP, producing a biocompatible MnO_x_/Ti_3_C_2_–SP composite with increased photothermal conversion efficiency of 30.6% [55]. The modification of Ta_4_C_3_ MXene by replacing the MnO_X_ with super-magnetic iron oxide nanoparticles (IONPs) further enhanced the *T*_*2*_-weighted MR imaging-guided PTT against cancer [56], while Ti_3_C_2_ MXene is functionalized by hydrophobic poly(lactic-*co*-glycolic acid) (PLGA) and SP, resulting in a PLGA/Ti_3_C_2_-SP nanocomposite which exhibited strong NIR absorption (808 nm) and high photothermal conversion [34]. The magnetic nanocomposite, functionalized with Fe_3_O_4_ nanocrystals, exhibited enhanced *T*_*2*_ relaxivity of 394.2 mM^−1^ s^−1^ for MR imaging and high photothermal conversion efficiency of 48.6% [32]. Other than tantalum and titanium carbides, niobium carbide has also been studied for its photothermal abilities, such as Nb_2_C nanosheets functionalized with biocompatible polyvinylpyrrolidone (PVP) [57]. The Nb_2_C nanocomposites featured a unique enzyme-responsive biodegradability to human myeloperixodase (hPMO), which generates hypochlorous acid (HOCl) and reactive radical intermediates, that contributes to polymeric or carbon-based materials’ degradation [88]. The PVP/Nb_2_C composite exhibited excellent photothermal conversion efficiency in both NIR-I and NIR-II biowindows (36.4% and 45.65%, respectively) [83]. Other than PVP, Nb_2_C nanosheets were also functionalized with cetanecyltrimethylammonium chloride (CTAC) to create a ‘therapeutic mesopore’ surface on the nanosheets, which exhibited a high drug loading capacity of 32.57% and inhibition efficiency of 92.37% against cancer cells [58].

#### MXene quantum dots (MQDs)

As discussed, MXenes are great biosensing tools due to its high electronic conductivity and ease to functionalize, however, standalone MXenes exhibit low photoluminescence (PL) response in aqueous environment, which limit their biological and optical applications.  In addition, some MXene nanosheets are still ‘too large’ for cell permeation, so it is essential for them to have strong PL and are of ultrasmall in size [59, 89]. The breakthrough is MXene quantum dots (MQDs). To date, MQDs have been employed for metal ion sensing, protein detection, electrocatalysis, and energy storage [90–96]. The first production of MQDs yield Ti_3_C_2_ MQDs for multicolour cellular imaging, through facile hydrothermal synthesis (100 and 120 °C)[59]. Another Ti_3_C_2_ MQDs were prepared through a fluorine-free synthesis technique which used mechanical force-assisted liquid exfoliation; bulk Ti_3_AlC_2_ was ultrasonically treated with a probe in tetrabutylammonium hydroxide (TBAOH) etching solution [60]. Ti_3_C_2_ MQDs exhibited large extinction coefficient of 52.8 Lg^−1^ cm^−1^ at NIR range (808 nm), which is ideal for  photothermal imaging. Also, they displayed high photothermal conversion efficiency of 52.2%, and showed great biocompatibility in vitro and in vivo [60]. Another study of Ti_2_N QDs exhibit excellent photothermal conversion efficiency of 48.62% and 45.51% in NIR-I and NIR-II biowindows with great biodegradable function, therefore, is best candidate as PA imaging-guide PTT agent [61]. MQDs can also be used as a fluorescence imaging probe, as example of fluorescence emission of Nb_2_C MQDs [62]. These MQDs were reported to display excellent chemical stability, biocompatibility, and possessed strong resistance to photobleaching [62].

Based on these theranostic studies of MXenes [49, 51, 52, 54], they show great potential as contrast agents in various bio-imaging and excellent therapeutic agents for PTT treatment. Most studied MXenes exhibited better photothermal conversion efficiency, compared to conventional Au nanorods [50]. Also, Ta-based MXene show great potential as MRI contrast agents [50], which can replace traditional Gd-based contrast agents that can destabilise and produce bare Gd^3+^ ions that are harmful to our bodies. Tantalum carbides can oxidize into tantalum oxides, but the systemic toxicity of tantalum oxide is quite low due to its poor solubility. Tantalum carbide is also comparable to Au nanoparticles as X-ray imaging contrast agent since they both possess similar X-ray attenuation coefficient. Overall, MXenes exhibit low cytotoxicity and good biodegradability for effective cancer treatment and safe human consumption.

#### Drug delivery

2D nanomaterials ranging from 1 to 100 nm which perfectly suit to the need of drug delivery system [97]. Due to their size, nanomaterials can travel more freely inside the human body, compared to larger molecules. Moreover, traditional drug administration requires high drug dosages for achieving therapeutic levels, which in turn caused toxicity to normal cells and tissues, and resistance of multiple drugs [98, 99]. This provide the motivation of nanomedicines as delivery agents in targeting specific tissues, by encapsulating or attaching to specified drugs [100]. MXenes have potential as great drug delivery systems, as their 2D planar structure and unique physicochemical properties could endorse them for drug loading for precision treatment [63]. Modified ultrathin Ti_3_C_2_ nanosheets are able to be transported easily throughout the blood vessels [34], and its large surface area enable efficient coating of anticancer drug doxorubicin (Dox) and show high drug-releasing percentages in acidic environment, leading to efficient eradication of the tumour [101]. Upon near-infrared (NIR) irradiation at 808 nm, the drug release percentage increased by 23.5% at very acidic condition [101].

To efficiently control the rate of drug release, MXene nanomaterials were integrated into cellulose hydrogel [64]. This type of MXene hydrogel shows the efficient releasing rate accelerated by NIR irradiation in addition to its illumination function for enlarging the pore dimensions of the composite hydrogel, accelerating the release of Dox [64]. Ti_3_C_2_/polyacrylamide (PAM) hydrogels polymerised of Ti_3_C_2_ nanosheets and acrylamide show better swelling properties as compared to traditional *N,N*-methylene bisacrylamide/polyacrylamide (BIS/PAM) hydrogel, effectively increasing the uptake of drug [65, 102]. The reason for the brilliant drug uptake in the MXene-composite hydrogel is aided by the hydrogen-bonding interactions between the water and hydrophilic surface functional groups of MXenes and the hydrophilic groups (-CONH_2_) of the polymer chains. The Ti_3_C_2_/PAM hydrogels tend to swell for a much longer time compared to BIS/PAM hydrogels, indicating a higher drug loading capacity [65]. Also, the drug-releasing percentage of Ti_3_C_2_/PAM hydrogels increased to 62.1–81.4%, compared to BIS/PAM hydrogels (45%).

Recently, magnetic nanomaterials have been introduced into drug delivery systems to increase the controllability of the nanocarriers especially under magnetic field [103, 104], example is combination of cobalt nanowires (CoNWs) and Ti_3_C_2_ nanosheets to form Ti_3_C_2_-CoNWs metal–semiconductor heterojunction [66]. The Ti_3_C_2_-based nanocarrier heterojunction exhibited an increased drug loading efficacy, with increasing Dox-nanocarrier ratios. At the highest mass ratio of 3, the DOX/Ti_3_C_2_-CoNWs nanocarrier heterojunction exhibited the highest drug loading capacity of 225.05%, which is much higher than single Ti_3_C_2_ nanosheets (84%) and most spherical drug delivery nanocarriers (10–30%) [105, 106]. Under acidic conditions, the solubility and the hydrophilicity of the Dox drug increase, prompting an acceleration of drug release [107]. Additionally, NIR irradiation of the Dox-loaded nanocarrier further increased the drug release percentage [66].

As studied, bare Ti_3_C_2_ MXene has been proven to be far superior for drug release compared to classic spherical systems, with an increase of > 50% in drug release percentage. Modifying the Ti_3_C_2_ nanosheets with hydrogel or magnetic semiconductors further improved drug release percentage by at least ~ 30% and ~ 200%, respectively. Hence, Ti_3_C_2_ MXene demonstrates a promising drug delivery nanocarrier, pristine or modified, for precision drug delivery and cancer treatment that is potentially way better than current nano-based drug delivery systems.

#### Antimicrobial activity

The high surface area to volume ratio and ease of surface functionalization has open a door for antimicrobial application to enable reaction with bacteria membranes [108]. Generally, nanomaterials do not trigger bacterial resistance due to their high membrane permeability, biocompatibility and potential for multiple antibacterial actions [108]. Prior to MXenes, the mode of action of graphene-based nanomaterials in antimicrobial application function by production of reactive oxygen species (ROS) and direct contact with bacteria membrane [109–113]. The first Ti_3_C_2_ MXene colloidal solution for its antibacterial properties need a high concentration dose of 200 μg mL^−1^ to give positive inhibition [67, 68]. Nevertheless, modified Ti_3_C_2_ modified with polyvinylidene fluoride (PVDF) greatly improve the antimicrobial activities [68].

Another fabrication of Ti_3_C_2_/chitosan composite nanofibers crosslinked with glutaraldehyde via electrospinning show more than 62% reduction of microbes [69]. Comparison of Ti_2_C and Ti_3_C_2_ MXenes against *E. coli* show that only Ti_3_C_2_ exhibit antibacterial properties [70], and the further examination of these proved that smaller-sized nanosheets demonstrated higher antibacterial activity [71]. In addition, the aggregation of nanomaterials due to its cationic charge was improved by combining Ti_3_C_2_ flakes with cationic polymeric poly-L-lysine (PLL), which changed the negative charge (− 5.6 mV) of Ti_3_C_2_ flakes to + 44.9 mV (1:1, PLL: Ti_3_C_2_) to extremely reduce the flocculation degree of the MXene flakes [22].

Another study has proposed that the unique features of MXene nanosheets that have sharp edges and small size can cut through the bacterial cell wall, which results in the release of bacteria DNA and eventually bacteria dispersion [71]. To date, the only study of antifungal properties of MXene was reported on Ti_3_C_2_ MXene on *Trichoderma reesei* (*T. reesei*) [20]. The results of this study demonstrated the inhibition of hyphae growth of *T. reesei* with the presence of delaminated MXene (d-Ti_3_C_2_), which exhibited similar results with antifungal medication amphotericin-B. The MXene nanosheets may also inhibit spore germination due to the sharp lamellar edges of the nanosheets. Hence, this study proved that Ti_3_C_2_ MXene can disrupt the life cycle of fungi and showed great potential of MXenes as promising antifungal agents.

Although the bacterial killing mechanism of MXenes are currently being investigated, the long-term colloidal stability of the MXene is still not fully understood. Generally, agglomeration or oxidation of 2D nanomaterials will cause the loss of its 2D characteristic structure and will directly affect their antimicrobial efficacy. Ideally, MXenes should be stable for long enough to induce its bactericidal properties, but also remain biocompatible. Hence, more research on improving the stability of MXene in physiological conditions should be carried out to increase its application as an antimicrobial agent.

## Conclusions and future outlooks

In this review, the general overview of structural organization of 2D MXenes is discussed. Various syntheses methods: top-down or bottom-up, fluorine-based or fluorine-free etching methods, are reviewed to produce biocompatible MXenes. Synthesised MXenes can then be further modified to enhance the biocompatibility/biodegradability and reduce the cytotoxicity of the 2D materials for specific biomedical applications. MXenes possess an exceptional potential for drug delivery, antimicrobial properties, tissue engineering, high surface area to volume ratio, as well as wide-ranging near-infrared absorption. These qualities make MXenes one of the most promising material for bio-applications. The chemistry of MXenes allows possible application in area that had not been explored such as surface coating of medical catheter, mask, and gloves because of their excellent antimicrobial activity. Flexibility and elasticity of MXenes have also been reported [114], mainly as thin films for electronic devices, which is also a suitable trait for surface coating as we do not want the surface of the catheter/ mask/gloves to harden. It is also possible to develop highly effective and non-invasive anticancer therapy because of their photodynamic/photo thermal chemotherapy synthetic effect. Nevertheless, research on the biocompatibility of MXene-based materials is still very limited, more attention should be focused on the systematic evaluation and adjustment of toxicity of MXene-based materials. For example, cell uptake behaviour, cytotoxicity mechanism, in vitro and in vivo MXenes should also be prudently investigated. Therefore, physiological effects of MXenes need to be fully understood, since MXene-based materials may accumulate in our body system, and long-term accumulation could lead to potential toxicity. Till date, reports on the interaction of MXene with human physiological system are not available. Previous findings of other 2D nanomaterials, such as graphene, on their behaviours in biological/physiological conditions can be referred to when conducting studies on MXenes. For example, graphene’s clearance behaviours can be referred to when studying the clearance pathway of MXenes, since this pathway is important to be fully understood for the materials to be deemed safe for clinical use. Prudent investigation of MXene for clinical use is an area that had not be explored. Also, more environmentally friendly preparation approaches need to be investigated to ensure minimal hazard to environment upon release. Through these studies, a rapid growth in the synthesis of new family of MXenes and their bright perspective in biomedical applications can be expected. This review showcases the wide range of applications of MXenes, their derivatives, and MXene-based composites in biosensors, cancer theranostics, cancer biomarkers drug delivery, and antimicrobial activity. We note that the use of MXenes in biomedical research is in its early stage, and systematic guidelines are required before biomedical applications of MXene-based materials can be achieved.

## Data Availability

Not applicable.
